# Diffuse Bilateral Pseudo-Cystic Pulmonary Lesions Revealing Metastatic Lung Adenocarcinoma: A Case Report and Literature Review

**DOI:** 10.7759/cureus.103730

**Published:** 2026-02-16

**Authors:** Nabil Tiresse, Abdelhalim Boucaid, raihana laamim, khalil chafi, Hicham Souhi

**Affiliations:** 1 Department of Pneumology, Mohamed VI Military Hospital, Dakhla, MAR; 2 Department of Pneumology, Mohamed V Military Training Hospital, Rabat, MAR; 3 Department of Diagnostic Radiology, Mohamed VI Military Hospital, Dakhla, MAR

**Keywords:** ct scan chest, diffuse cystic lung disease, metastatic adenocarcinoma of lung, molecular targeted therapy, pet scans, pseudo-cystic pulmonary lesions

## Abstract

Diffuse cystic lung diseases are a heterogeneous group of disorders with multiple etiologies, including rare malignant causes. We report the case of a 49-year-old non-smoking man presenting with a two-month history of dry cough without hemoptysis, dyspnea, or chest pain. Chest computed tomography (CT) revealed diffuse bilateral pseudo-cystic pulmonary lesions and scattered nodules. Bone window analysis identified osteolytic lesions of the sternum and the eighth thoracic vertebra. Additional CT scans demonstrated multiple secondary lesions involving the brain, spine, and left iliac bone. 18F-fluorodeoxyglucose positron emission tomography (FDG-PET) showed hypermetabolic pulmonary, mediastinal, and skeletal lesions. Brain and spinal magnetic resonance imaging confirmed multiple cerebral and vertebral secondary localizations, with perimedullary tumoral epiduritis. A CT-guided biopsy of the sternal lytic lesion established the diagnosis of poorly differentiated lung adenocarcinoma. Tumor cells expressed thyroid transcription factor-1 and cytokeratin AE1/AE3, with programmed death-ligand 1 expression <1%. Molecular analysis revealed no mutations in EGFR, KRAS, BRAF, HER2, or MET. Systemic chemotherapy with carboplatin and pemetrexed was initiated, with pembrolizumab planned, and radiotherapy was administered for the vertebral lesion.

This case highlights a rare pseudo-cystic radiological presentation of metastatic lung adenocarcinoma and underscores the importance of considering a neoplastic etiology in patients presenting with diffuse bilateral thin-walled pulmonary lesions.

## Introduction

Diffuse cystic lung diseases comprise a heterogeneous group of disorders with diverse etiologies, including infectious, inflammatory, genetic, and, more rarely, malignant causes including metastatic sarcomas, angiosarcoma, and, more rarely primary lung adenocarcinoma [1]. Lung cancer presenting with diffuse cystic or pseudo-cystic pulmonary lesions is an uncommon radiological manifestation that may lead to diagnostic delay [2].

We report the case of a patient diagnosed with lung adenocarcinoma revealed by diffuse bilateral pseudo-cystic pulmonary lesions. This rare presentation highlights the importance of considering an underlying neoplastic process when confronted with diffuse cystic lung abnormalities.

## Case presentation

A 49-year-old non-smoking man with no significant past medical history was referred to the pulmonology department for a two-month history of persistent dry cough. There was no associated sputum production, hemoptysis, chest pain, or dyspnea. Physical examination, including pleuropulmonary assessment, revealed no bronchial crackles, and the remainder of the examination was unremarkable.

**Figure 1:**
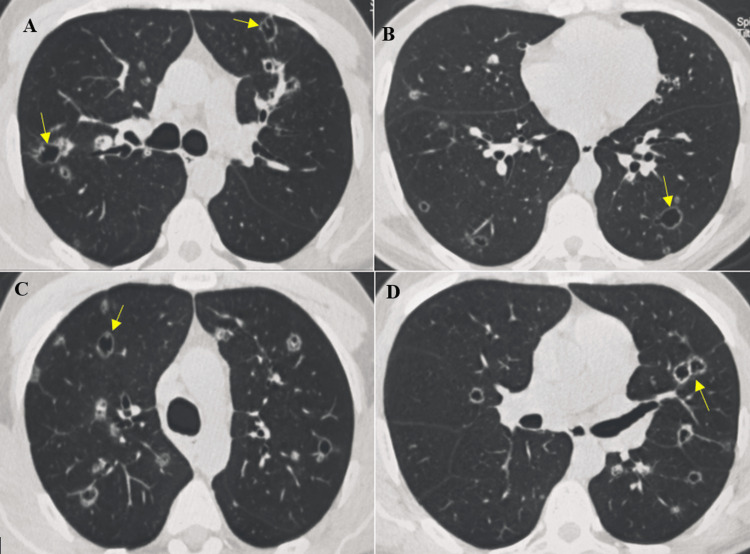
Axial chest computed tomography (CT) images (parenchymal window) A: CT scan showing two pseudocystic lesions in the two upper lobes, right and left (yellow arrow) B: Pseudocystic lesions with a wall < 2mm in the bilateral posterobasal region (yellow arrow) C: Ground-glass nodules in the right subpleural upper lobe with other upper bilateral pseudocystic lesions (yellow arrow) D: Left posterobasal nodule with several ipsilateral pseudocystic lesions and one contralateral lesion (yellow arrow)

A frontal chest radiograph showed multiple bilateral thin-walled lucent lesions. Chest computed tomography (CT) revealed diffuse bilateral air-filled lesions with thin, occasionally irregular walls measuring less than 2 mm in thickness (Figures 1A, 1B), giving them a pseudocystic appearance. No internal fluid levels were observed. Multiple disseminated bilateral nodules and micronodules were also identified, ranging from 2 to 10 mm in diameter (Figures 1C, 1D). Their distribution was random in both lungs, without zonal predominance, and no centrilobular or perilymphatic pattern was identified.

**Figure 2:**
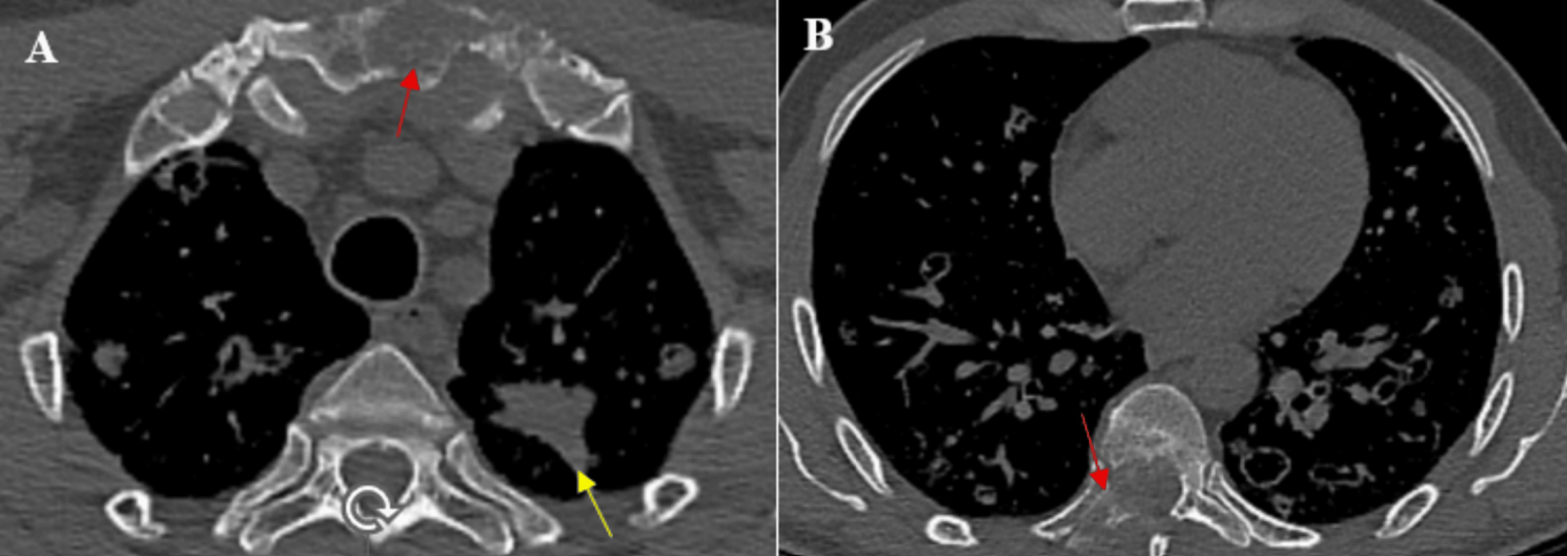
Axial chest CT images (bone window) (A) Osteolytic lesion of the sternal manubrium (red arrow) associated with left upper lobe consolidation (yellow arrow). (B) Osteolytic lesion involving the eighth thoracic vertebra (red arrow). On both axial sections, multiple bilateral pseudo-cystic pulmonary lesions are also observed.

Bone window analysis demonstrated a well-defined osteolytic lesion of the sternum with cortical destruction but no significant soft-tissue mass associated with left upper lobe consolidation (Figure 2A), and an additional osteolytic lesion centered in the body of the eighth thoracic vertebra (T8), extending to the ipsilateral transverse process. There was a cortical breakthrough with posterior epidural extension into the spinal canal, forming an endocanalar soft-tissue component responsible for spinal cord compression (Figure 2B). Although precise measurements were not recorded at the time of initial imaging, the lesion was considered clinically significant given the extent of bone destruction and epidural involvement. Based on the radiological pattern, a neoplastic etiology was initially suspected, given the random distribution of nodules and the presence of associated osteolytic lesions. Septic emboli were considered because of the multiple bilateral nodular lesions; however, the absence of fever, negative infectious workup, and a normal transthoracic echocardiography made this diagnosis less likely. Pulmonary Langerhans cell histiocytosis was also discussed, but the lack of upper lobe predominance and the patient’s non-smoking status argued against this hypothesis.

Additional cerebral and abdominopelvic CT scans revealed multiple secondary lesions, including a small right parietal cortical enhancing nodule measuring 5 mm in diameter, without associated vasogenic edema or mass effect, as well as a lytic lesion involving both the cortical and medullary bone of the left iliac wing.

Subsequent brain and spinal magnetic resonance imaging (MRI) confirmed the 5-mm right parietal lesion and showed no evidence of brain herniation or additional significant intracranial mass effect. MRI also revealed a lytic lesion of the eighth thoracic vertebra with perimedullary tumoral epiduritis, without spinal cord signal abnormality.

**Figure 3:**
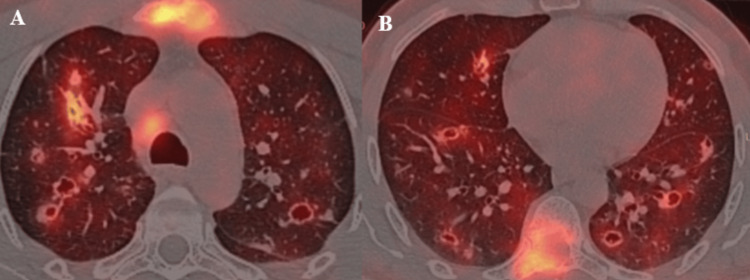
Axial thoracic positron emission tomography (PET) images (A) Intense pathological hypermetabolic uptake involving the sternum associated with pathological mediastinal lymph node hypermetabolism. (B) Intense pathological hypermetabolic uptake involving the eighth thoracic vertebra. Both images also demonstrate increased metabolic activity within the pulmonary parenchymal lesions.

18F-fluorodeoxyglucose positron emission tomography (FDG-PET) demonstrated intense hypermetabolism of the bilateral pulmonary lesions, associated with hypermetabolic mediastinal and left supraclavicular lymphadenopathy, as well as hypermetabolic osteolytic lesions of the sternum (Figure 3A), the right side of the eighth thoracic vertebra (Figure 3B), and the left iliac bone. 

**Figure 4:**
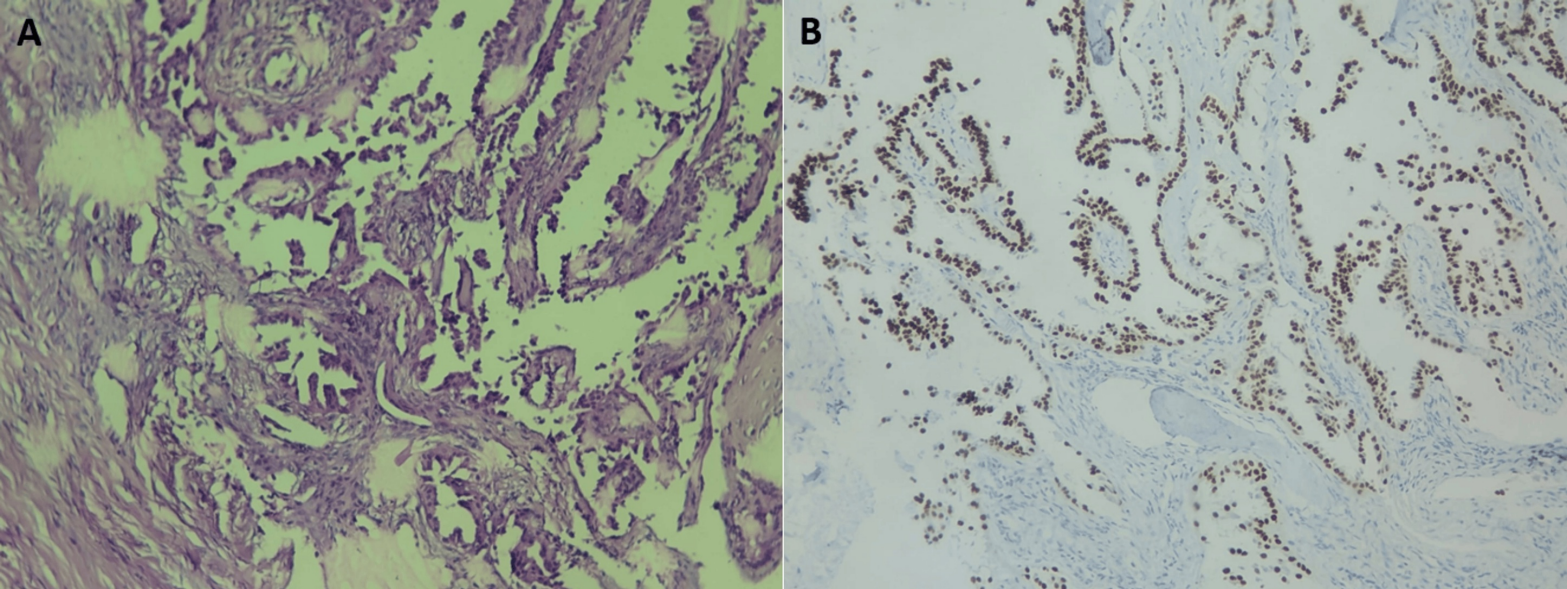
Histopathological and immunohistochemical findings of the sternal bone metastasis from lung adenocarcinoma A: Bone tissue infiltrated by a malignant epithelial proliferation arranged in papillary and trabecular patterns. Hematoxylin and eosin staining (H&E), original magnification ×100. B: Immunohistochemical staining demonstrates tumor cell expression of thyroid transcription factor-1 (TTF-1), supporting pulmonary origin. Original magnification ×100.

A CT-guided percutaneous biopsy of the sternal lytic lesion confirmed the diagnosis of metastatic poorly differentiated adenocarcinoma consistent with pulmonary origin (Figure 4A). Immunohistochemical analysis demonstrated strong tumor cell expression of thyroid transcription factor-1 (TTF-1) (Figure 4B) and cytokeratin AE1/AE3, with no expression of synaptophysin, supporting a primary lung adenocarcinoma and excluding neuroendocrine differentiation. Given the unequivocal TTF-1 positivity, additional mucin staining was not performed. Tumor cells showed no expression of programmed death-ligand 1 (PD-L1) or anaplastic lymphoma kinase (ALK). Molecular analysis revealed no mutations in EGFR, KRAS, BRAF, HER2, or MET genes. ROS1 and RET fusion testing were negative, while NTRK fusion analysis was not performed.

Systemic chemotherapy based on carboplatin and pemetrexed was initiated. The first two cycles were well tolerated. The introduction of pembrolizumab is currently under consideration. Palliative radiotherapy was administered to the eighth thoracic vertebra because of the risk of spinal cord compression.

## Discussion

Diffuse cystic lung diseases constitute a heterogeneous group of disorders characterized by the presence of spherical or irregular thin-walled air-filled spaces within the pulmonary parenchyma [1,3]. They may result from a wide range of etiologies, most commonly including pulmonary Langerhans cell histiocytosis, infectious diseases such as Pneumocystis jirovecii pneumonia, lymphangioleiomyomatosis, and various interstitial lung diseases. Malignant causes are much rarer but should be considered in the appropriate clinical and radiological context [3]. Among neoplasms associated with cystic pulmonary lesions are gastrointestinal carcinomas, mesenchymal tumors, sarcomas, and, more infrequently, primary lung adenocarcinomas [2].

Several mechanisms have been proposed to explain the development of cystic or cavitary lesions in lung cancer. One of the most widely accepted hypotheses involves partial obstruction of the terminal bronchioles by tumor cell infiltration, creating a check-valve mechanism. This leads to distal air trapping, inflammatory changes, progressive destruction of alveolar septa, and subsequent dilatation of small airways [2,4]. Cystic lesions should be distinguished from thick-walled, irregular cavities, which are more commonly observed in pulmonary metastases from adenocarcinomas [2]. These cystic abnormalities may also be complicated by spontaneous pneumothorax [3-5].

Identifying a malignant etiology in diffuse cystic lung disease remains challenging, particularly in patients with minimal or nonspecific respiratory symptoms and in the setting of numerous benign differential diagnoses, including histiocytosis and infectious disorders. This diagnostic ambiguity may result in empiric antibiotic therapy and subsequent delays in establishing the correct diagnosis [2,5]. In our patient, bilateral pseudocystic lesions involving multiple pulmonary levels initially favored non-malignant diagnoses, especially given a clinical presentation that was not strongly suggestive of an underlying neoplastic process (Figures 1A-1D). Nevertheless, the diagnosis was established at a relatively early stage. A meticulous review of the bone window on chest computed tomography revealed osteolytic lesions of the sternum and vertebrae, prompting comprehensive staging and histopathological confirmation obtained through a CT-guided sternal biopsy (Figures 2A, 2B).

Chest CT plays a pivotal role in both diagnosis and staging of cystic lung diseases. It allows identification of well-demarcated, rounded radiolucent lesions of variable size with thin walls, clearly separated from the surrounding normal lung parenchyma [2,3]. In addition, CT facilitates the detection of associated pulmonary, mediastinal, and osseous lesions and guides histopathological sampling. Importantly, rapid enlargement or an increasing number of cystic lesions on follow-up CT should raise suspicion for an underlying invasive pulmonary carcinoma [6,7].

FDG-PET remains the cornerstone of staging, providing a comprehensive assessment of metabolically active pulmonary, nodal, and distant metastatic lesions [5]. In our case, FDG-PET further completed the staging work-up by demonstrating pathological hypermetabolic uptake in multiple suspected metastatic sites, including the sternum and the thoracic vertebra (Figures 3A, 3B).

Brain and spinal magnetic resonance imaging are essential for detecting secondary cerebral and vertebral localizations and play a crucial role in planning radiotherapy, particularly in cases with threatening spinal involvement [7]. Some authors have also reported the potential contribution of bronchoalveolar lavage cytology in detecting malignant cells, although its diagnostic yield remains variable [7].

Molecular characterization of tumor cells is a key step in the management of lung adenocarcinoma, particularly in non-smoking patients. In metastatic lung adenocarcinoma occurring in non-smokers, systematic testing for driver mutations, including EGFR, ALK, KRAS, BRAF, and HER2, is essential to guide targeted therapies. Evaluation of PD-L1 expression is also required to assess eligibility for immunotherapy [8]. The identification of EGFR mutations, for example, has been associated with improved prognosis in patients treated with tyrosine kinase inhibitors such as afatinib [7].

Overall, the prognosis of metastatic lung adenocarcinoma presenting with diffuse bilateral cystic lesions remains poor in most reported cases, particularly in the absence of actionable molecular alterations. The KEYNOTE-189 trial demonstrated a significant overall survival benefit with the combination of platinum-based chemotherapy, pemetrexed, and pembrolizumab in patients with metastatic nonsquamous non-small cell lung cancer, regardless of PD-L1 expression, including tumors with PD-L1 <1% [9]. In our case, no actionable driver mutations were identified, and PD-L1 expression was negative; this regimen would have represented a standard first-line therapeutic option. However, treatment decisions must also take into account real-world factors such as performance status, comorbidities, and potential contraindications to immunotherapy.

This case is limited by its single-patient nature; however, it highlights an uncommon radiological presentation of lung adenocarcinoma and emphasizes the importance of careful imaging analysis in diffuse cystic lung diseases.

## Conclusions

This case highlights an uncommon radiological presentation of lung cancer, which remains one of the leading causes of cancer-related mortality worldwide. A neoplastic etiology should be systematically considered among the primary differential diagnoses in patients presenting with diffuse bilateral pseudo-cystic pulmonary lesions. Comprehensive imaging assessment, histopathological confirmation, and molecular profiling are essential to ensure timely diagnosis and optimize therapeutic management.
